# Supplementary material for: Effectiveness of Positive Deviance, an Asset-Based Behavior Change Approach, to Improve Knowledge, Attitudes, and Practices Regarding Dengue in Low-Income Communities (Slums) of Islamabad, Pakistan: A Mixed-Method Study
Source: Insects. 2022 Jan 8;13(1):71. doi: 10.3390/insects13010071 (PMC8780378; doi:10.3390/insects13010071)
Supplement: Supplementary file 1 [file insects-13-00071-s001.zip › insects-1453481-supplementary.pdf]

Supplementary file

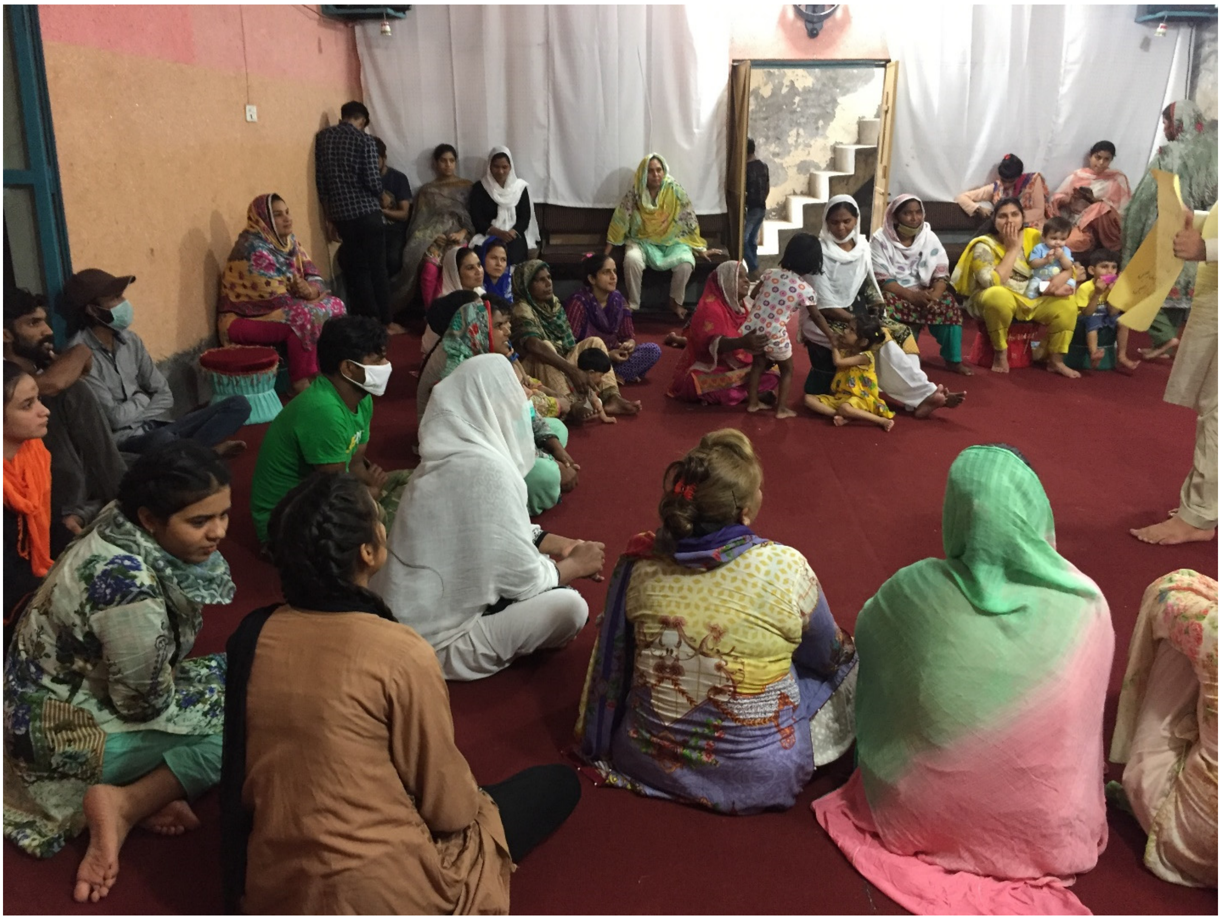

Figure S1. Community sensitization meeting with key community stakeholders.

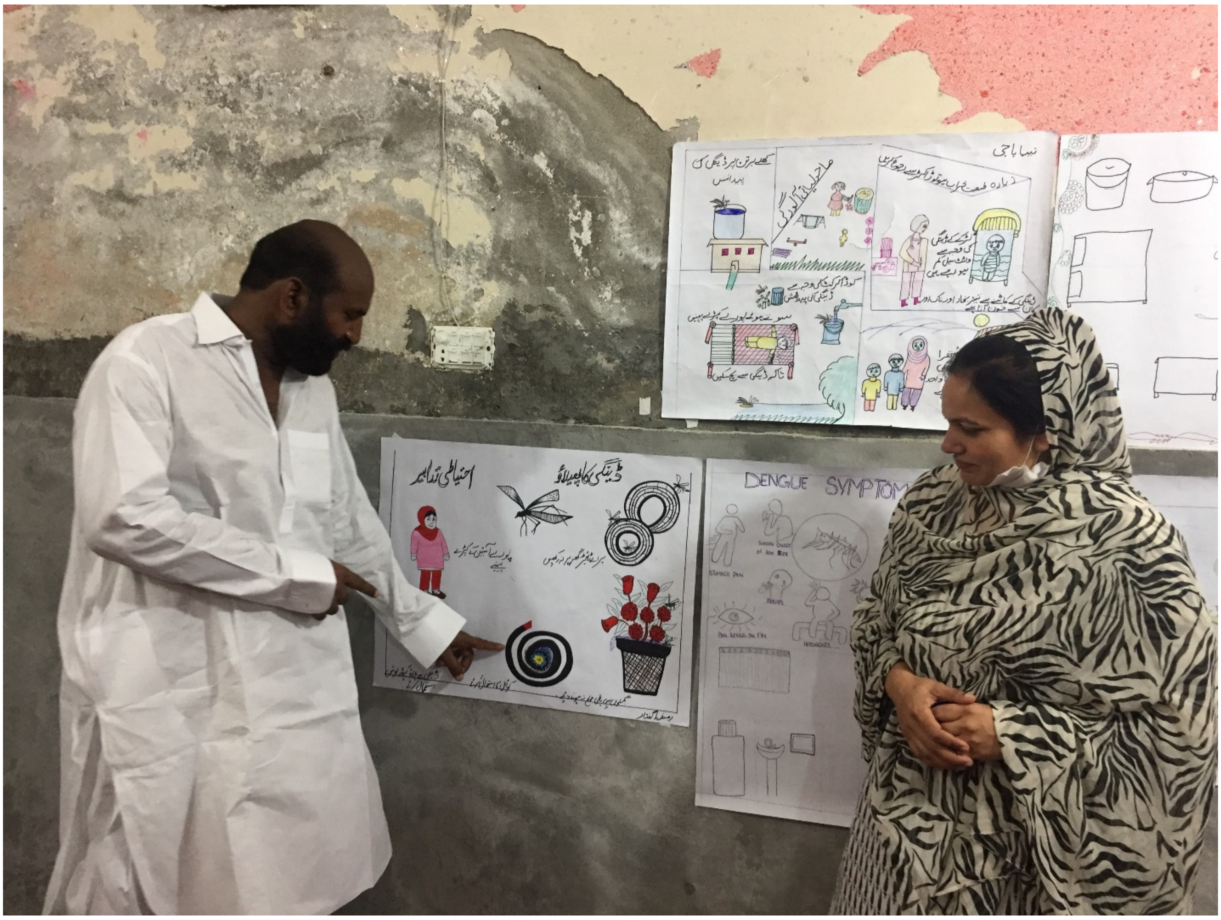

Figure S2. PD role models share their positive behaviors during the feedback session.

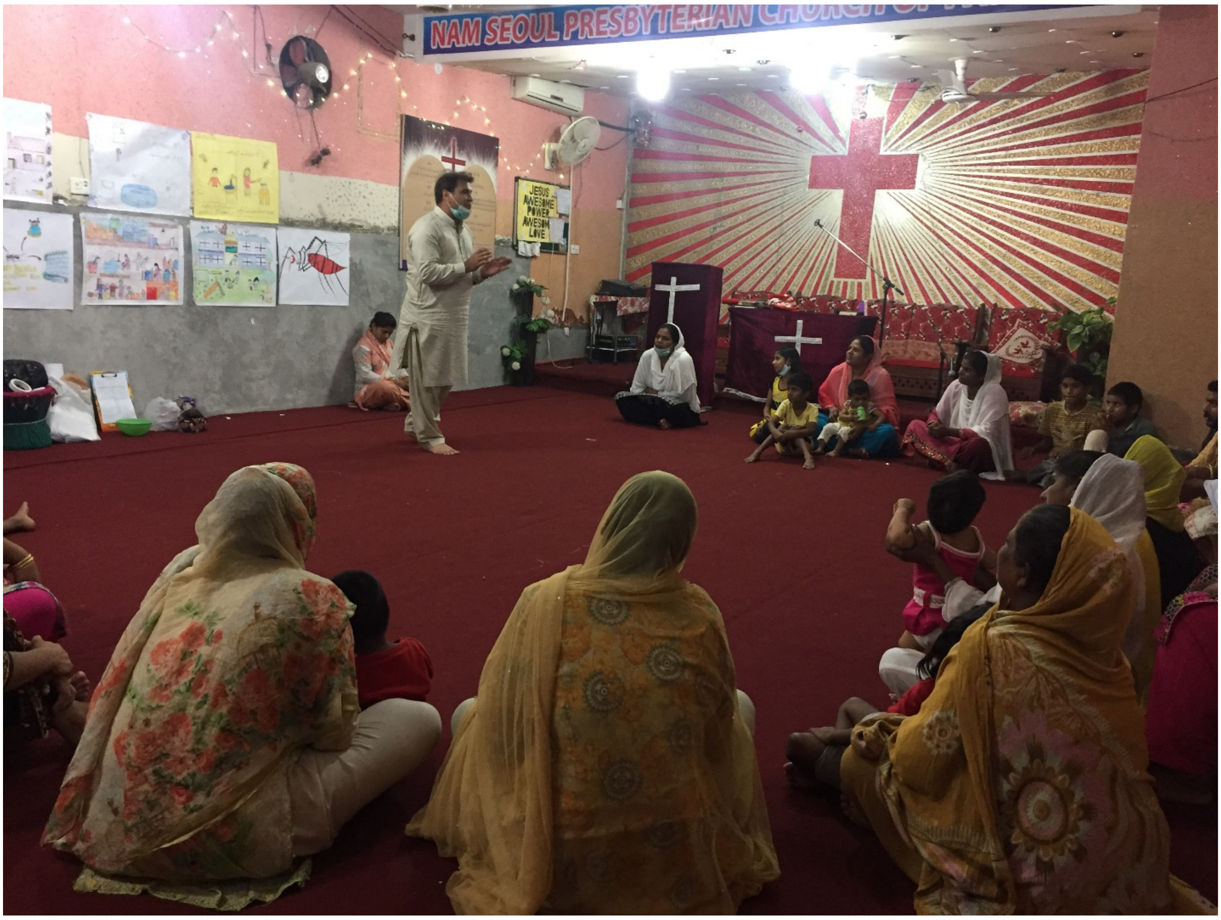

Figure S3. PD health education session with female community members.

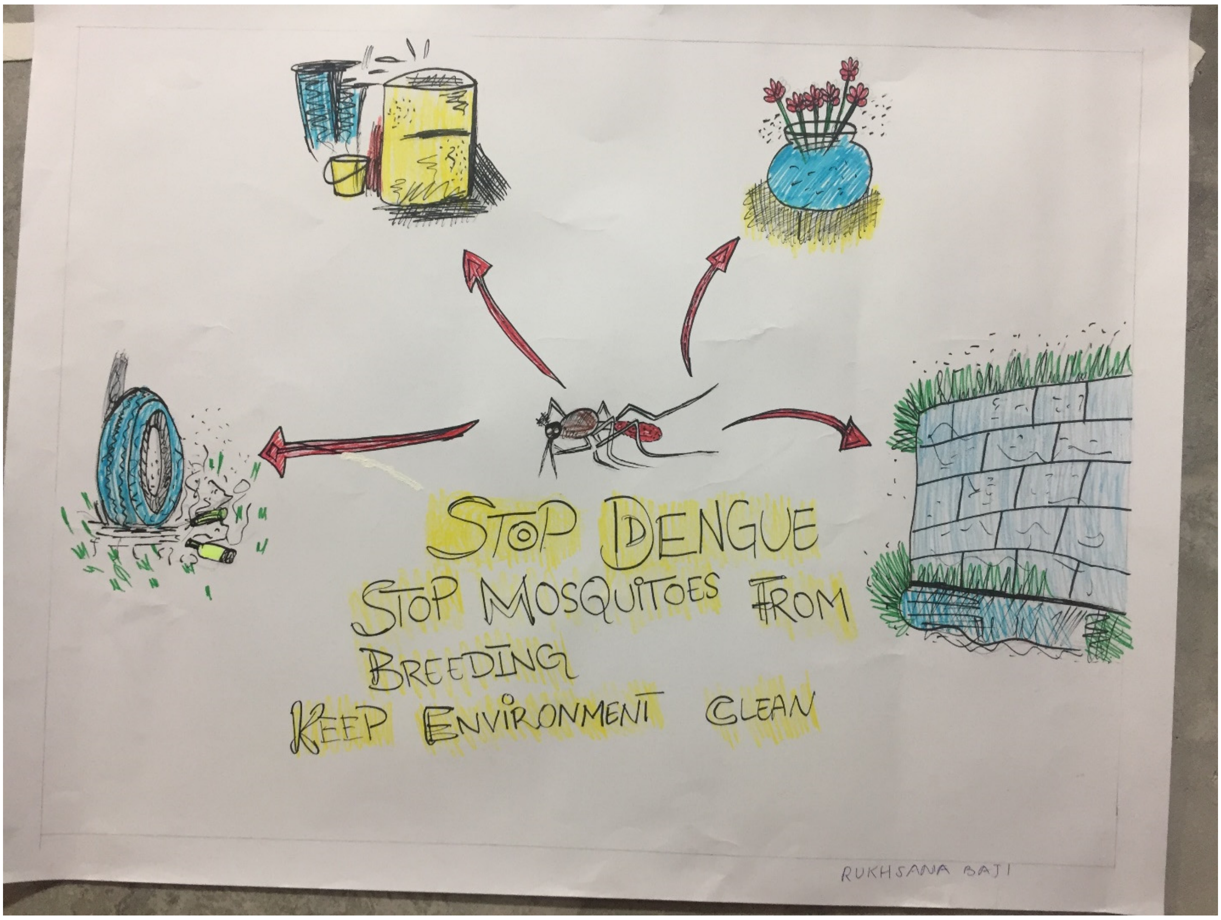

Figure S4. An illustration made by a community member for the sketch competition.
